# Supplementary material for: Self-Reported Practices in Opioid Management of Chronic Noncancer Pain: An Updated Survey of Canadian Family Physicians
Source: J Clin Med. 2020 Oct 14;9(10):3304. doi: 10.3390/jcm9103304 (PMC7602479; doi:10.3390/jcm9103304)

Table S1. Rating of factors affecting decision not to prescribe strong opioids for chronic noncancer pain (shown in decreasing order of importance in 2018 survey).

		Rating *, %			Total Responses, *n*
		**Not Important**	**Neutral**	**Important**	
Concern about long-term adverse effects, eg, addiction or misuse	2010	5	5	88	57
	2018	6	8	88	40
Strong opioids are commonly diverted and abused in community	2010	7	7	83	57
	2018	8	13	75	40
Lack of evidence for effectiveness of strong opioids in CNCP	2010	21	21	47	57
	2018	8†	13	73†	40
Concern about becoming a "target prescriber" of opioids	2010	23	12	60	57
	2018	26	5	65	40
Concern about short-term adverse effects	2010	35	28	32	57
	2018	28	18	55	40
Concern about audit from regulatory or monitoring body	2010	47	18	32	57
	2018	28†	18	51 †	40
Takes too much time to titrate and monitor	2010	63	12	16	57
	2018	55	15	30 †	40
Inadequate knowledge of dosages of strong opioids	2010	55	13	24	55
	2018	68†	5	20	40

* Per cent of respondents rating importance of factor as 1 or 2 (not important), 3 (neutral), or 4 or 5 (important) on 5-point Likert scale. † Difference is 10% or larger. Percentage may not total 100% because some respondents indicated 'no opinion'. The total of eligible participants to answer this question is 43 in 2018 ("I only prescribe weak opioids for CNCP").

Figure S1. Responses to the open-ended question "At what daily dose of morphine or equivalent do you consider that patients might need to be referred for a second opinion?"

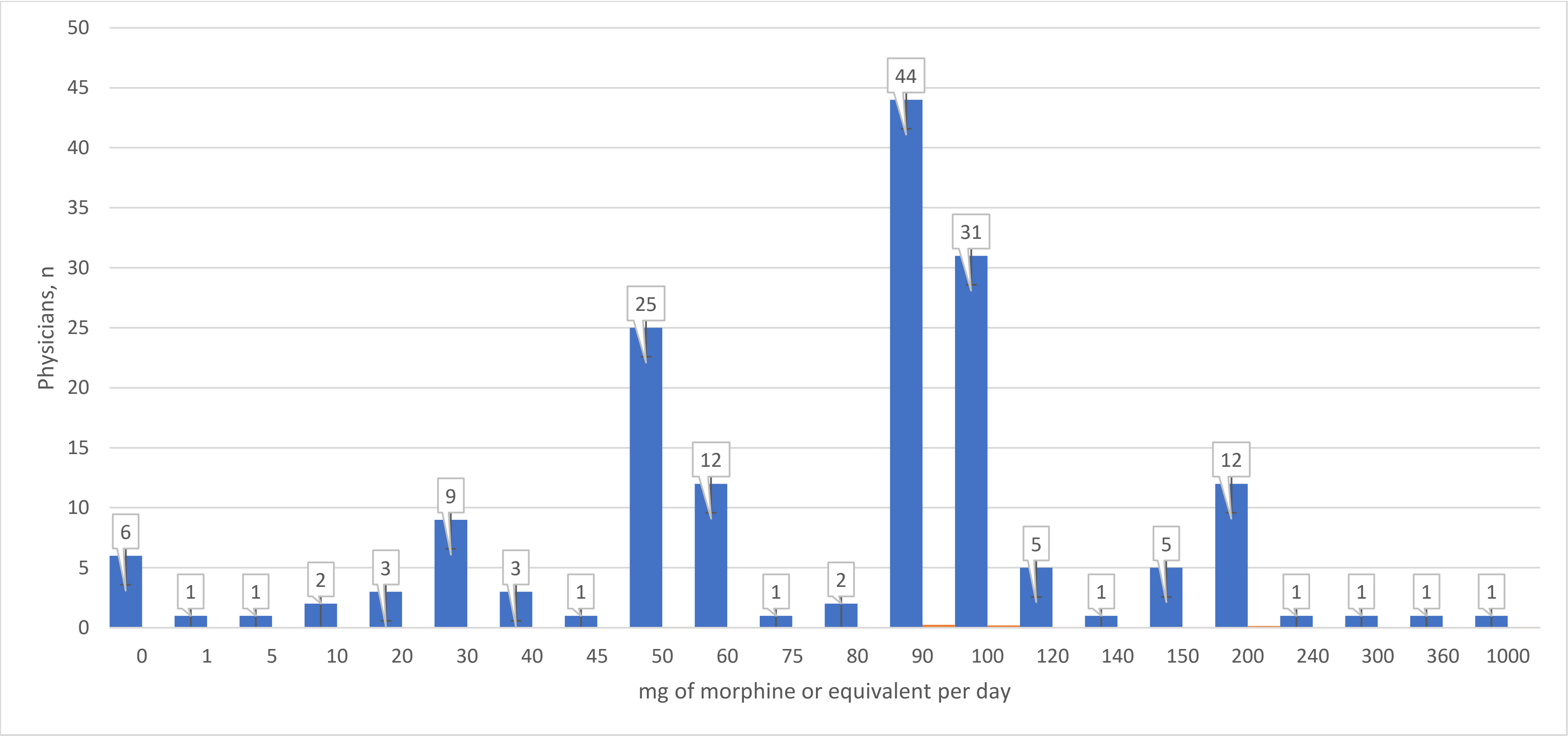

Supplement: Supplementary file 1 [file jcm-09-03304-s001.zip › jcm-912519-supplementary materials/jcm-912519-Supplementary-table & figure.pdf]
