# Supplementary material for: Self-Reported Practices in Opioid Management of Chronic Noncancer Pain: An Updated Survey of Canadian Family Physicians
Source: J Clin Med. 2020 Oct 14;9(10):3304. doi: 10.3390/jcm9103304 (PMC7602479; doi:10.3390/jcm9103304)
Supplement: Supplementary file 1 [file jcm-09-03304-s001.zip › jcm-912519-supplementary materials/Supplementary Materials S1 - Survey of Canadian Family Physicians about treatment of patients with chronic pain.pdf]

2018 Survey of Canadian Family Physicians about treatment of patients with chronic pain

This survey is for family physicians who treat patients with chronic non-cancer pain (CNCP). If you do not fit this category please do not complete the survey.

Results of this survey will help develop educational programs about treatment of Chronic Non Cancer Pain.

Completion of this survey is voluntary, and neither the researchers nor the colleges will be able to identify those who responded or not responded the survey.

Completion of this survey will take approximately 15 minutes.

Consent:

- **There are minimal risks for completing the survey, though you may feel discomfort in disclosing your opinions and experiences.**
- **You can withdraw at any time before submitting the survey simply by closing the browser, the data from non-submitted surveys will not be saved. Once you submit the survey you won't be able to withdraw from the study.**
- **Once you start the survey you are not allowed to skip questions.**
- **Your responses are anonymous and will not be linked to you.**
- **Data will be aggregated and reported by province, and rural/urban categories; your individual data will not be reported.**
- **Data will be compared with past surveys, and may be compared with future results if the survey is repeated in a few years.**
- **Data may be used as part of a master thesis.**
- **If you want results of the study, please contact a member of the research team: Dr. Andrea Furlan at andrea.furlan@utoronto.ca, or Dr. Angela Carol at acarol@cpso.on.ca or Santana Díaz at santana.diaz@utoronto.ca**
- **Survey data will be stored on an encrypted USB drive and kept for 7 years. After that the USB drive will be destroyed.**
- **If you have any questions please contact the principal investigator Dr. Andrea Furlan at andrea.furlan@utoronto.ca**
- **Completion of the survey implies your consent to participate.**
- **If you want to keep a copy of this consent form for your records please feel free to print a copy.**

This study has been approved by the University of Toronto Research Ethics Board.

* 1. **Please rate your confidence in prescribing opioids for Chronic non-cancer pain:**

1 Not very confident	2	3	4	5 Very confident
☆	☆	☆	☆	☆

* 2. **Which of the following definitions of chronic non-cancer pain is MOST similar to YOUR definition?**

- ◯ Pain that persists more than 3 MONTHS
- ◯ Pain that persists more than 6 MONTHS
- ◯ Pain persisting beyond the time normally associated with healing for a specific illness or injury

For the remainder of the survey, please respond according to the definition of Chronic Non-Cancer Pain YOU use in your practice

* 3. **Do you prescribe weak or strong opioids for patients with Chronic Non-Cancer Pain (CNCP)?**

- Weak opioids- Codeine, Tramadol, Propoxyphene, Meperidine, Pentazocine
- Strong opioids -Morphine, Oxycodone, Hydromorphone, Fentanyl patch, Methadone

- ◯ I do NOT prescribe opioids for CNCP
- ◯ I prescribe only WEAK opioids for CNCP
- ◯ I prescribe only STRONG opioids for CNCP
- ◯ I prescribe WEAK and STRONG opioids for CNCP

* 4. Please indicate how important each of the following is in your decision NOT to prescribe opioids for patients with Chronic Non-Cancer Pain (CNCP)?

	1 Not very important	2	3	4	5 Very important	No opinion
A. Takes too much time to titrate and monitor	○	○	○	○	○	○
B. Inadequate knowledge of which opioids to use	○	○	○	○	○	○
C. Inadequate knowledge of dosages	○	○	○	○	○	○
D. Concern about short-term adverse effects	○	○	○	○	○	○
E. Concern about long-term adverse effects like addiction and misuse	○	○	○	○	○	○
F. Concern about audit from regulatory or monitoring body	○	○	○	○	○	○
G. Concern that patients complain of pain out of proportion to objective findings	○	○	○	○	○	○
H. Lack of evidence for effectiveness of opioids in CNCP	○	○	○	○	○	○
I. Type of practice	○	○	○	○	○	○
J. Concern about becoming a "target prescriber" of opioids	○	○	○	○	○	○

If you wold like to mention other factors or make comments, please enter below:

5. Skip to the final part of the survey

○ Yes

* 6. Please indicate how important each of the following is in your decision to prescribe only WEAK opioids for patients with Chronic Non-Cancer Pain.

	1. Not very important	2	3	4	5. Very important	No opinion
A. Takes too much time to titrate and monitor	○	○	○	○	○	○
B. Inadequate knowledge of dosages of which opioids to use	○	○	○	○	○	○
C. Inadequate knowledge of dosages of strong opioids	○	○	○	○	○	○
D. Concern about short-term adverse effects	○	○	○	○	○	○
E. Concern about long-term adverse effects like addiction and misuse	○	○	○	○	○	○
F. Concern about audit from regulatory or monitoring body	○	○	○	○	○	○
G. Lack of evidence for effectiveness of strong opioids in CNCP	○	○	○	○	○	○
H. Strong opioids commonly diverted and abused in community	○	○	○	○	○	○
I. Concern about becoming a "target prescriber" of opioids	○	○	○	○	○	○

If you would like to mention other factors or make comments, please enter below.

* 7. BEFORE STARTING opioid therapy, in what percentage of your patients with Chronic Non-Cancer Pain do you do the following?

	Never	Less than 25% of patients	Less than 50% of patients	More than 50% of patients	More than 75% of patients	Always
A. Assess patient's level of pain intensity using a scale	○	○	○	○	○	○
B. Assess patient's level of function (e.g. social, recreational, occupational)	○	○	○	○	○	○
C. Assess risk of addiction using screening tool	○	○	○	○	○	○
D. Assess for past/current substance use disorders as well as active psychiatric disorders.	○	○	○	○	○	○
E. Conduct formal psychological screening	○	○	○	○	○	○
F. Do urine drug screening	○	○	○	○	○	○
G. Have patient sign a treatment agreement	○	○	○	○	○	○
H. Explain potential benefits of long-term opioid therapy	○	○	○	○	○	○
I. Explain potential harms of long-term opioid therapy	○	○	○	○	○	○
J. If patient is on a benzodiazepine, try to taper them off	○	○	○	○	○	○
K. Give the patient written information about opioid therapy	○	○	○	○	○	○
L. Refer to colleague for assessment	○	○	○	○	○	○
M. Confirm that the patient has a condition that has been shown to benefit from opioids	○	○	○	○	○	○

	Never	Less than 25% of patients	Less than 50% of patients	More than 50% of patients	More than 75% of patients	Always
N. Beginning opioid therapy be sure to prescribe a dose less than 50mg morphine equivalents daily	○	○	○	○	○	○

If you have any comments please enter them below

* 8. WHILE MONITORING opioid therapy, in what percentage of your patients with Chronic Non-Cancer Pain do you do the following?

	Never	Less than 25% of patients	Less than 50% of patients	More than 50% of patients	More than 75% of patients	Always
A. Assess patient's level of pain intensity using a scale	○	○	○	○	○	○
B. Assess patient's level of function (e.g. social, recreational, occupational)	○	○	○	○	○	○
C. Observe for aberrant drug-related behavior such as requesting higher doses or accessing opioids from other sources	○	○	○	○	○	○
D. Do routine or urine drug screening	○	○	○	○	○	○
E. Assess for specific adverse effects (e.g. nausea, constipation, drowsiness, dizziness)	○	○	○	○	○	○
F. If patient has unacceptable side effects, try a different opioid	○	○	○	○	○	○
G. If patient has unacceptable side effects try a lower dose	○	○	○	○	○	○
H. If patient has insufficient pain relief, increase the dose	○	○	○	○	○	○
I. If patient has insufficient pain relief, try a different opioid	○	○	○	○	○	○
J. If patient has insufficient pain relief, taper off opioid and try another modality	○	○	○	○	○	○
K. Ask patient to bring remaining medication to check compliance with the prescription	○	○	○	○	○	○

	Never	Less than 25% of patients	Less than 50% of patients	More than 50% of patients	More than 75% of patients	Always
L.If patient is experiencing a serious challenge in tapering, try a referral to a formal multidisciplinary program						
M. Advise the patient to use caution while driving or operating machinery.						

If you have any comments please enter them below

* 9. Please rate how useful or not useful the following factors would be in helping you optimize you management of patients with chronic non-cancer pain on opioids

	1 Not very useful	2	3	4	5 Very useful	No opinion
A. Validated screening tool to screen patients for risk of addiction						
B. Tips in recognizing patients at high risk of addiction						
C. Availability of urine drug screening at local lab						
D. Knowledge of practical aspects of urine drug screening (e.g., collection sample, interpreting results)						
E. Validated scale to assess pain intensity						
F. Validated scale to assess function (e.g. social, recreational, functional)						
G. Knowledge of risks and benefits of different opioids						
H. Up to date guideline on use of opioids in CNCP						

	1 Not very useful	2	3	4	5 Very useful	No opinion
I. CME in optimal use of opioids in CNCP	○	○	○	○	○	○
J. Readily available help, such as physician mentor or 1-800 help line	○	○	○	○	○	○
K. Access to patients' opioid prescription history from provincial monitoring program	○	○	○	○	○	○
L. Patient education material	○	○	○	○	○	○
M. Improved access to consultants who are experts in pain or addiction	○	○	○	○	○	○
O. Availability of non pharmacological options	○	○	○	○	○	○
P. Accesibility of other pharmacological agents (butrans/transdermal buprenorphine)	○	○	○	○	○	○

If you have any comments please enter them below

* 10. Please indicate whether you agree or disagree with the following statements.

	Disagree	Agree	No opinion
A. There is evidence from randomized controlled trials that opioids are effective in short-term (up to 3 months) relief of CNCP	○	○	○
B. There is evidence from randomized controlled trials that opioids are effective in long-term (over 3 months) relief of CNCP	○	○	○
C. Some strong opioids provide greater pain relief that others	○	○	○

	Disagree	Agree	No opinion
D. Some strong opioids are more likely to lead to addiction than others	○	○	○
E. Patients may safely be switched from a high dose of codeine to a fentanyl patch	○	○	○
F. Controlled-release opioids have a lower risk of addiction than immediate release opioids	○	○	○
G.Controlled-release opioids are more effective in controlling pain than immediate-realease opioids	○	○	○
H. A 30% reduction in pain intensity is considered clinically significant	○	○	○
I. Pain relief is a more important indicator of opioid effectiveness than functional ability	○	○	○
J. Opioid replacement therapy is effective for patients with opioid abuse disorder	○	○	○
K. Medical cannabis is effective for neuropathic pain	○	○	○

If you have any comments please enter them below

* 11. At what daily dose of morphine or equivalent do you consider that patients might need to be referred for a second opinion?

mg of morphine or equivalent per day

* 12. What is the MINIMUM daily dose of opioid in morphine equivalents that your patient would be taking before you would prescribe FENTANYL patch?

- ◯ Fentanyl is my first line opioid
- ◯ 20 morphine equivalents
- ◯ 40 morphine equivalents
- ◯ 60 morphine equivalents
- ◯ No minimum dose, varies with patient condition
- ◯ No opinion

* 13. For approximately how many patients per month do you write prescriptions for WEAK opioids for Chronic Non-Cancer Pain?

- Weak opioids are Codeine, Tramadol, Propoxyphene, Meperidine, Pentazocine

- ◯ 0 to 5 patients per month
- ◯ 6 to 10 patients per month
- ◯ 11 to 20 patients per month
- ◯ more than 20 patients per month

* 14. For approximately how many patients per month do you write prescriptions for STRONG opioids for Chronic Non-Cancer Pain?

- Strong opioids are Morphine, Oxycodone, Hydromorphone, Fentanyl patch, Methadone

- ◯ 0 to 5 patients per month
- ◯ 6 to 10 patients per month
- ◯ 11 to 20 patients per month
- ◯ more than 20 patients per month

* 15. What type of health care professional are you?

- ◯ Family physician
- ◯ Specialist physician
- ◯ Nurse Practitioner
- ◯ Other health care professional

Please specify

[ ]

* 16. What is your gender?

- ◯ Female
- ◯ Male
- ◯ Prefer no to answer

* 17. What year did you start practicing as a health professional?

Year: [ ]

* 18. Have you had any advanced training in pain management such as a diploma course or clinical traineeship?

- ◯ Yes
- ◯ No

* 19. We would like to know how busy your practice is. Approximately how many patients in TOTAL do you see in your office or outpatient clinic per month?

Patients per month:

* 20. What is the size of the community in which you practice?

- Under 5,000 people
- 5,000 to 25,000 people
- 25,000 to 100,000 people
- 100,000 to 500,000 people
- More than 500,000 people

* 21. What is the waiting time for your patients to see a PAIN specialist for a NON-URGENT referral?

- Less than 1 month
- 1 to 6 months
- 6 to 12 months
- More than 12 months
- I don't know
- I don't have this available

* 22. What is the waiting time for your patient for a referral to a colleague for a second opinion regarding the possibility of increasing the dose to more than 90mg morphine equivalents daily?

- Less than a month
- 1 to 6 months
- 6 to 12 months
- More than 12 months
- I don't know
- I don't have this available

* 23. What is the waiting time for your patients to see an ADDICTION specialist for a NON-URGENT referral?

- Less than 1 month
- 1 to 6 months
- 6 to 12 months
- More than 12 months
- I don't know
- I don't have this available

* 24. In what province do you spend most of your time practicing?

- Alberta
- British Columbia
- Manitoba
- New Brunswick
- Newfoundland and Labrador
- Northwest Territories
- Nova Scotia
- Nunavut
- Ontario
- Prince Edward Island
- Quebec
- Saskatchewan
- Yukon

* 25. The first three characters of your postal code at work indicate whether you practice in a rural or urban setting.
Is the second character of your postal code a zero?

- ◯ Yes
- ◯ No
