# Supplementary material for: Self-Reported Practices in Opioid Management of Chronic Noncancer Pain: An Updated Survey of Canadian Family Physicians
Source: J Clin Med. 2020 Oct 14;9(10):3304. doi: 10.3390/jcm9103304 (PMC7602479; doi:10.3390/jcm9103304)
Supplement: Supplementary file 1 [file jcm-09-03304-s001.zip › jcm-912519-supplementary materials/Supplementary Materials S2 - Sondage aupres des medecins de famille canadiens.pdf]

Bienvenue au: Sondage auprés des médecins de famille canadiens

Cette enquête s'adresse aux médecins de famille qui traitent des patients souffrant de douleur chronique non cancéreuse. Si vous ne correspondez pas à cette catégorie, veuillez ne pas remplir ce questionnaire. Les résultats de cette enquête aideront à développer des programmes éducatifs sur le traitement de la douleur chronique non cancéreuse. Ce sondage est volontaire, et ni les chercheurs ni les collèges ne seront en mesure d'identifier ceux qui ont répondu (ou non) à l'enquête.
Cette enquête prendra environs 15 minutes à remplir.

Consentement :

- **Les risques de répondre à ce sondage sont minimes, mais vous pourriez être mal à l'aise d'exprimer vos opinions et expériences.**
- **Vous pouvez vous retirer en tout temps avant de transmettre le sondage simplement en fermant votre navigateur. Les données des sondages non transmis ne seront pas sauvegardées. Une fois que vous aurez transmis le sondage, vous ne pourrez pas vous retirer de l'étude.**
- **Une fois que vous commencez à répondre au sondage, vous ne pouvez pas ignorer de questions.**
- **Vos réponses sont anonymes et ne permettront pas de vous identifier.**
- **Les données seront regroupées et présentées par province et selon des catégories rurales et urbaines; vos données individuelles ne seront pas divulguées.**
- **Les données seront comparées à celles des sondages antérieurs, et pourraient être comparées à des résultats futurs si le sondage est de nouveau réalisé dans quelques années.**
- **Les données pourraient être utilisées dans le cadre d'une thèse de maîtrise.**
- **Si vous désirez obtenir les résultats de l'enquette veuillez communiquer avec un membre de l'équipe de recherche : Dre Andrea Furlan à l'adresse andrea.furlan@utoronto.ca, Dre Angela Carol à l'adresse acarol@cpso.on.ca et Santana Díaz S. à l'adresse santana.diaz@utoronto.ca**
- **Les données du sondage seront conservées sur une clé USB chiffrée et conservées pendant sept ans. Au bout des sept années, la clé USB sera détruite.**
- **Ce projet a fait l'objet d'un examen de la part du Comité d'éthique de la recherche de l'Université de Toronto.**
- **Si vous avez des questions, veuillez communiquer avec la chercheuse principale, Dre Andrea Furlan, à l'adresse andrea.furlan@utoronto.ca**
- **Le fait de répondre au sondage signifie que vous consentez à y participer.**
- **Si vous désirez conserver une copie de ce formulaire de consentement pour vos dossiers, n'hésitez pas à en imprimer une copie.**

* 1. Veuillez indiquer votre niveau de confiance au moment de prescrire des opioïdes pour une douleur chronique non cancéreuse

1 Pas très confiant	2	3	4	5 Très confiant
☆	☆	☆	☆	☆

* 2. Laquelle des définitions ci-dessous de douleur chronique non cancéreuse correspond le PLUS à la définition que VOUS en avez?

- ◯ Une douleur qui persiste pendant plus de 3 MOIS
- ◯ Une douleur qui persiste pendant plus de 6 MOIS
- ◯ Une douleur qui persiste au-delà de la période normalement associée au rétablissement pour une maladie ou une blessure en particulier.

-Pour le reste du sondage, veuillez répondre en fonction de la définition de douleur chronique non cancéreuse que VOUS utilisez dans le cadre de votre exercice.

* 3. * Prescrivez-vous des opioïdes faibles ou forts à des patients souffrant de douleur chronique non cancéreuse (DCNC)?

-Opioïdes faibles: codéine, tramadol, propoxyphène, mépéridine, pentazocine
-Opioïdes forts: morphine, oxycodone, hydromorphone, timbre de fentanyl, méthadone

- ◯ Je ne prescris PAS d'opioïdes pour la DCNC
- ◯ Je prescris des opioïdes FAIBLES seulement pour la DCNC
- ◯ Je prescris des opioïdes FORTS seulement pour la DCNC
- ◯ Je prescris des opioïdes FAIBLES et FORTS pour la DCNC

* 4. Veuillez indiquer dans quelle mesure chacun des éléments ci-dessous pèse dans votre décision de ne PAS prescrire d'opioïdes à des patients souffrant de douleur chronique non cancéreuse (DCNC).

	1 Pas très important	2	3	4	5 Très important	Pas d'opinion
A. Trop long à doser et à surveiller	○	○	○	○	○	○
B. Connaissance inadéquate des opioïdes à utiliser	○	○	○	○	○	○
C. Connaissance inadéquate des doses	○	○	○	○	○	○
D. Préoccupation liée aux effets indésirables à court terme.	○	○	○	○	○	○
E. Préoccupation liée aux effets indésirables à long terme comme la dépendance et la mauvaise utilisation	○	○	○	○	○	○
F. Crainte d'une vérification de la part d'un ordre des médecins ou d'un organisme de surveillance	○	○	○	○	○	○
G. Crainte que les patients se plaignent de douleur hors de proportion comparativement aux conclusions objectives	○	○	○	○	○	○
H. Manque de données probantes concernant l'efficacité des opioïdes pour traiter la DCNC	○	○	○	○	○	○
I. Type de genre pratique médicale	○	○	○	○	○	○
J. Crainte de devenir un « prescripteur cible » d'opioïdes	○	○	○	○	○	○

Si vous désirez mentionner d'autres facteurs ou formuler des commentaires, veuillez le faire ci-dessous.

5. Aller à la dernière partie de l'enquête

- ◯ Oui

* 6. Veuillez indiquer dans quelle mesure chacun des éléments ci-dessous pèse dans votre décision de prescrire des opioïdes FAIBLES seulement à des patients souffrant de douleur chronique non cancéreuse.

	1. Pas très important	2	3	4	5. Très important	Pas d'opinion
A. Trop long à doser et à surveiller	○	○	○	○	○	○
B. Connaissance inadéquate des doses et des opioïdes à utiliser	○	○	○	○	○	○
C. Connaissance inadéquate des doses d'opioïdes forts	○	○	○	○	○	○
D. Préoccupation liée aux effets indésirables à court terme.	○	○	○	○	○	○
E.Préoccupation liée aux effets indésirables à long terme comme la dépendance et la mauvaise utilisation	○	○	○	○	○	○
F. Crainte d'une vérification de la part d'un ordre des médecins ou d'un organisme de surveillance	○	○	○	○	○	○
G. Manque de données probantes concernant l'efficacité des opioïdes forts pour traiter la DCNC	○	○	○	○	○	○
H. Les opioïdes forts sont couramment détournés et font l'objet d'abus dans la communanté	○	○	○	○	○	○
I. A.Crainte de devenir un « prescripteur cible » d'opioïdes	○	○	○	○	○	○

Si vous désirez mentionner d'autres facteurs ou formuler des commentaires, veuillez le faire ci-dessous

* 7. AVANT DE DÉMARRER un traitement par opioïdes, chez quel pourcentage de vos patients souffrant de douleur chronique non cancéreuse faites-vous ce qui suit?

	Jamais	Moins de 25 % des patients	Moins de 50 % des patients	Plus de 50 % des patients	Plus de 75 % des patients	Toujours
A. Évaluer le niveau d'intensité de la douleur du patient à l'aide d'une échelle	◯	◯	◯	◯	◯	◯
B. Évaluer le niveau de fonctionnement du patient (p. ex., social, dans les loisirs, au travail)	◯	◯	◯	◯	◯	◯
C. Évaluer le risque de dépendance à l'aide d'un outil de dépistage	◯	◯	◯	◯	◯	◯
D. Vérifier la presence actuelle ou passé de tout troubles de toxicomanie ainsi que les troubles psychiatriques actifs	◯	◯	◯	◯	◯	◯
E. Réaliser un examen psychologique officiel	◯	◯	◯	◯	◯	◯
F. Réaliser un test de dépistage de drogue dans l'urine	◯	◯	◯	◯	◯	◯
G. Demander au patient de signer une entente de traitement	◯	◯	◯	◯	◯	◯
H. Expliquer les avantages possibles d'un traitement de longue durée par opioïdes	◯	◯	◯	◯	◯	◯
I.Expliquer les effets secondaires ou risques possibles d'un traitement de longue durée par opioïdes	◯	◯	◯	◯	◯	◯
J. Si le patient suit un traitement par benzodiazépine, essayer de diminuer graduellement la dose à zero	◯	◯	◯	◯	◯	◯

	Jamais	Moins de 25 % des patients	Moins de 50 % des patients	Plus de 50 % des patients	Plus de 75 % des patients	Toujours
K. Remettre au patient des renseignements écrits concernant un traitement par opioïdes	○	○	○	○	○	○
L. Aiguiller vers un collège pour une évaluation	○	○	○	○	○	○
M. Confirmer que le patient souffre d'une affection pour laquelle un traitement par opioïdes a démontré de bons résultats	○	○	○	○	○	○
N. Au début du traitement par opioïdes, s'assurer de prescrire une dose quotidienne inférieure à 50 mg en équivalents morphine	○	○	○	○	○	○

Si vous avez des commentaires, veuillez les ignore ci-dessous

* 8. PENDANT LA SURVEILLANCE d'un traitement par opioïdes, chez quel pourcentage de vos patients souffrant de douleur chronique non cancéreuse faites-vous ce qui suit?

	Jamais	Moins de 25 % des patients	Moins de 50 % des patients	Plus de 50 % des patients	Plus de 75 % des patients	Toujours
A. Évaluer le niveau d'intensité de la douleur du patient à l'aide d'une échelle	○	○	○	○	○	○
B. Évaluer le niveau de fonctionnement du patient (p. ex., social, dans les loisirs, au travail)	○	○	○	○	○	○
C. Surveiller pour un comportement anormal lié au médicament, comme des plaintes agressives pour des doses plus élevées ou de se procurer des opioïdes d'autres sources	○	○	○	○	○	○
D. Réaliser un test de dépistage de drogue dans l'urine	○	○	○	○	○	○
E. Vérifier la présence d'effets indésirables particuliers, (p. ex., nausée, constipation, somnolence, étourdissements)	○	○	○	○	○	○
F. Si le patient présente des effets secondaires inacceptables, essayer un opioïde différent	○	○	○	○	○	○
G. Si le patient présente des effets secondaires inacceptables, essayer une dose moins forte	○	○	○	○	○	○
H. Si le soulagement de la douleur du patient est insuffisant, augmenter la dose	○	○	○	○	○	○
I. Si le soulagement de la douleur du patient est insuffisant, essayer un opioïde différent	○	○	○	○	○	○

	Jamais	Moins de 25 % des patients	Moins de 50 % des patients	Plus de 50 % des patients	Plus de 75 % des patients	Toujours
J. Si le soulagement de la douleur du patient est insuffisant, diminuer graduellement la dose de l'opioïde et essayer une autre forme de traitement.	○	○	○	○	○	○
K. Demander au patient de rapporter la partie inutilisée du médicament pour vérifier le respect de l'ordonnance	○	○	○	○	○	○
L. Si le patient éprouve beaucoup de difficulté à diminuer graduellement la dose, essayer un aiguillage vers un programme multidisciplinaire officiel.	○	○	○	○	○	○
M. Informer le patient de faire preuve de prudence au moment de conduire ou de se servir de machinerie	○	○	○	○	○	○

Si vous avez des commentaires, veuillez les saisir ci-dessous.

* 9. Veuillez indiquer dans quelle mesure les facteurs ci-dessous seraient utiles ou non pour vous aider à optimiser votre gestion des patients souffrant de douleur chronique non cancéreuse qui prennent des opioïdes

	1 Pas très utile	2	3	4	5 Très utile	Pas d'opinion
A. Outil de dépistage validé pour vérifier quels sont les patients à risque de dépendance	○	○	○	○	○	○
B. Conseils pour reconnaître les patients à risque élevé de dépendance	○	○	○	○	○	○
C. Accessibilité d'un test de dépistage de drogue dans l'urine au laboratoire local	○	○	○	○	○	○

	1 Pas très utile	2	3	4	5 Très utile	Pas d'opinion
D. Connaissance des aspects pratiques d'un test de dépistage de drogue dans l'urine, (p. ex., collecte d'échantillons, interprétation des résultats).						
E. Échelle validée pour évaluer l'intensité de la douleur						
F. Échelle validée pour évaluer le fonctionnement, (p. ex., social, dans les loisirs, fonctionnel).						
G. Connaissance des risques et des avantages de différents opioïdes						
H. Lignes directrices à jour sur l'utilisation des opioïdes pour traiter la DCNC						
I. EMC en utilisation optimale des opioïdes pour traiter la DCNC						
J. Aide facilement accessible, par exemple un médecin mentor ou une ligne d'aide 1 800						
K. Accès aux antécédents d'ordonnances d'opioïdes des patients provenant d'un programme provincial de surveillance						
L. Document éducatif à l'intention des patients						
M. Meilleur accès à des conseillers experts en douleur ou en dépendance						
O. Disponibilité d'options non pharmacologiques						

	1 Pas très utile	2	3	4	5 Très utile	Pas d'opinion
P. Accessibilité d'autres agents pharmacologiques (BuTrans/timbre transdermique de buprénorphine)	○	○	○	○	○	○

Si vous avez des commentaires, veuillez les ignore ci-dessous.

* 10. Veuillez indiquer si vous êtes en accord ou en désaccord avec les énoncés suivants

	En désaccord	En accord	Pas d'opinion
A. Des essais contrôlés à répartition aléatoire démontrent que les opioïdes sont efficaces pour le soulagement à court terme (jusqu'à concurrence de 3 mois) de la DCNC	○	○	○
B. Des essais contrôlés à répartition aléatoire démontrent que les opioïdes sont efficaces pour le soulagement à long terme (plus de 3 mois) de la DCNC	○	○	○
C. Certains opioïdes forts offrent un plus grand soulagement de la douleur que d'autres	○	○	○
D. Certains opioïdes forts sont plus susceptibles que d'autres de créer une dépendance	○	○	○
E. Il pourrait être possible de faire passer les patients d'une dose élevée de codéine à un timbre de fentanyl de façon sécuritaire	○	○	○

	En désaccord	En accord	Pas d'opinion
F. Les opioïdes à libération contrôlée présentent un risque moins élevé de dépendance que les opioïdes à libération immédiate	○	○	○
G. Les opioïdes à libération contrôlée sont plus efficaces dans le contrôle de la douleur que les opioïdes à libération immédiate	○	○	○
H. Une diminution de l'ordre de 30 % de l'intensité de la douleur est considérée comme étant importante au plan clinique	○	○	○
I. Le soulagement de la douleur est un indicateur plus important de l'efficacité d'un opioïde que la capacité fonctionnelle	○	○	○
J. Le traitement de substitution aux opioïdes est efficace chez les patients souffrant d'un trouble de dépendance aux opioïdes	○	○	○
K. Le cannabis thérapeutique est efficace dans le soulagement des douleurs neuropathiques	○	○	○

Si vous avez des commentaires, veuillez les ignore ci-dessous

* 11. À quelle dose quotidienne de morphine ou d'un équivalent considérez-vous que les patients auraient besoin d'être aiguillés pour obtenir un second avis?

mg de morphine au d'un équivalent per jour:

* 12. À environ combien de patients par mois prescrivez-vous des opioïdes FAIBLES pour une douleur chronique non cancéreuse?
-Les opioïdes faibles sont la codéine, le tramadol, le propoxyphène, la mépéridine et la pentazocin

- 0 à 5 patients par mois
- 6 à 10 patients par mois
- 11 à 20 patients par mois
- Plus de 20 patients par mois

* 13. Quelle serait la dose quotidienne MINIMALE d'opioïdes exprimée en équivalent morphine que devrait prendre votre patient avant que vous lui prescriviez un timbre de FENTANYL?

- Le fentanyl est mon opioïde de première ligne
- 20 équivalents morphine
- 40 équivalents morphine
- 60 équivalents morphine
- Aucune dose minimale, varie selon l'état du patient
- Pas d'opinion

* 14. * À environ combien de patients par mois prescrivez-vous des opioïdes FORTS pour une douleur chronique non cancéreuse?

-Les opioïdes forts sont la morphine, l'oxycodone, l'hydromorphone, les timbres de fentanyl, la méthadone

- ◯ 0 à 5 patients par mois
- ◯ 6 à 10 patients par mois
- ◯ 1 à 20 patients par mois
- ◯ Plus de 20 patients par mois

* 15. Quel type de professionnel de la santé êtes-vous?

- ◯ Médecin de famille
- ◯ Médecin spécialiste
- ◯ Membre du personnel infirmier praticien
- ◯ Autre professionnel de la santé

Veuillez préciser

* 16. Quel est votre sexe?

- ◯ Femme
- ◯ Homme
- ◯ Je préfère ne pas répondre.

* 17. En quelle année avez-vous commencé à exercer en tant que professionnel de la santé?

Année :

* 18. Avez-vous déjà suivi une formation avancée en gestion de la douleur comme un cours d'études supérieures ou un stage clinique?

- ◯ Oui
- ◯ Non

* 19. Nous aimerions savoir dans quelle mesure votre cabinet est fréquenté. Environ combien de patients au TOTAL voyez-vous à votre cabinet ou clinique externe par mois?

Patients par mois :

* 20. Quelle est la taille de la collectivité dans laquelle vous exercez?

- ○ Moins de 5 000 habitants
- ○ De 5 000 à 25 000 habitants
- ○ De 25 000 à 100 000 habitants
- ○ De 100 000 à 500 000 habitants
- ○ Plus de 500 000 habitants

* 21. Combien de temps vos patients doivent-ils attendre avant de consulter un spécialiste de la DOULEUR pour un aiguillage NON URGENT?

- ○ Moins d'un mois
- ○ 1 à 6 mois
- ○ 6 à 12 mois
- ○ Plus de 12 mois
- ○ Je ne sais pas.
- ○ Je ne possède pas cette information

* 22. Combien de temps vos patients doivent-ils attendre lorsque vous les aiguillez vers un collègue pour obtenir un deuxième avis concernant la possibilité d'augmenter la dose quotidienne à plus de 90 mg en équivalents morphine?

- ○ Moins d'un mois
- ○ 1 à 6 mois
- ○ 6 à 12 mois
- ○ Plus de 12 mois
- ○ Je ne sais pas.
- ○ Je ne possède pas cette information

* 23. Combien de temps vos patients doivent-ils attendre avant de consulter un spécialiste des DÉPENDANCES pour un aiguillage NON URGENT?

- ○ Moins d'un mois
- ○ 1 à 6 mois
- ○ 6 à 12 mois
- ○ Plus de 12 mois
- ○ Je ne sais pas.
- ○ Je ne possède pas cette information

* 24. Dans quelle province exercez-vous la majeure partie du temps?

- Alberta
- Colombie-Britannique
- Île-du-Prince-Édouard
- Manitoba
- Nouveau-Brunswick
- Nouvelle-Écosse
- Nunavut
- Ontario
- Québec
- Saskatchewan
- Terre-Neuve-et-Labrador
- Territoires du Nord-Ouest
- Yukon

* 25. Les trois premiers caractères de votre code postal au travail indiquent si vous exercez en milieu rural ou urbain.
Le deuxième caractère de votre code postal est-il un zéro?

- Oui
- Non
